# Editorial: New concepts and techniques to treat peri-trochanteric fracture

**DOI:** 10.3389/fsurg.2024.1545562

**Published:** 2025-01-30

**Authors:** Jixing Fan, Yang Lv

**Affiliations:** ^1^ Department of Orthopedics, Peking University Third Hospital, Beijing, China; ^2^ Engineering Research Center of Bone and Joint Precision Medicine, Ministry of Education, Beijing, China

**Keywords:** hip fracture, lateral femoral wall, surgical technique, cost-Effectiveness, TAD

**Editorial on the Research Topic**
New concepts and techniques to treat peri-trochanteric fracture

The number of hip fractures increases quickly with the aging of the global population and the concomitant rise in life expectancy in recent decades, which have high mortality, morbidity and healthcare costs. An early surgical procedure is recommended for these patients, enabling early mobilization and avoiding bed-rest complications. Despite great efforts made by orthopedic trauma surgeons, postoperative complications remain one of the major challenges after surgery for hip fractures, which might be related to the patients' frailty, surgical delay, surgical technique, the choice of the internal fixator and the placement of implant. This research topic aims to provide a collection of reports to present novel concepts and techniques on the treatment of hip fractures for avoiding postoperative complications.

The lateral femoral wall (LFW), which is initially described by Gotfried, provides a lateral buttress for sliding of the head-neck fragment. It has been reported that LFW fracture is the primary independent predictor of fixation failure complication which needed reoperation. Although substantial evidence has proven that the nail itself could play the role of a lateral buttress and prevent excessive sliding of the head-neck fragment, intramedullary fixation encounters great difficulties in reducing or fixing the LFW when it is broken, which might increase the instability of unstable intertrochanteric fractures. Although a few additional fixations have been used to reconstruct the broken lateral wall, there are still some challenges to determine which method is the best choice. The article by Zhao et al. evaluates the clinical outcome of intramedullary nail supplemented by buttress plate or cable in the treatment of intertrochanteric fracture with broken lateral wall. Their results show that augmentation of intramedullary nailing system using plate/cable contributes to reconstructing the lateral femur wall.

Hip fractures in elderly patients are serious injuries that can lead to immobility and death. Early surgery is thought to play an important role regarding survival. Guidelines from the United States, United Kingdom and Canada recommend surgery within 24 to 48 h after admission. However, these recommendations are still discussed controversially. Some researchers argue that early surgery can lead to an increased risk of perioperative complications, including pneumonia, deep venous thrombosis, bleeding, pulmonary embolism and so on. Forssten et al. demonstrate that patients with hip fractures who are frail or have a higher cardiac risk suffer from an increased risk of in-hospital mortality when surgery is postponed >24 h. This study involves a total of 254,400 patients. This study finds that delaying surgery beyond 24 h from admission increases the risk of mortality for all geriatric hip fracture patients. The magnitude of the negative impact increases with the patient's level of cardiac risk and frailty.

Intertrochanteric femoral fractures are common hip fractures among elderly individuals who usually have multiple systemic comorbidities such as cardiovascular, respiratory, and endocrine conditions. Therefore, minimizing surgical trauma, avoiding complex procedures, reducing surgical duration, and accomplishing fracture reduction and fixation using the simplest and most practical methods play a crucial role in the rapid recovery of elderly patients. The study by Huang et al. explores the clinical efficacy of intramedullary reduction techniques for irreducible intertrochanteric fractures with negative medial cortical support. This retrospective analysis involved 69 patients and divided them into experimental group (36 cases) and control group (33 cases). The study concludes that intramedullary reduction techniques used during surgery demonstrate simplicity, significant reduction in surgical duration, decreased intraoperative bleeding, fewer amounts of intraoperative fluoroscopy, improved fracture reduction quality, and reduced surgical complexity for irreducible intertrochanteric femoral fractures with negative medial cortical support. Similarly, Wu et al. introduces a “3–2–1” body surface localization method preoperatively to avoid incorrect incision positioning and improve the operation efficiency and reduction quality in intertrochanteric fractures. This novel surgical technique can predict the incisions for the needle insertion point, spiral blade, and locking nails, create minimally invasive incisions, avoid incorrect incision position, facilitate accurate intraoperative intramedullary nail placement, reduce the incision size, intraoperative bleeding, and radiation exposure.

Nondisplaced or impacted femoral neck fractures constitute a substantial portion of hip fractures, accounting for approximately 15% of the overall hip fracture burden. Whether internal fixation (IF) or hemiarthroplasty (HA) is the optimal treatment strategy remains controversial for these nondisplaced femoral neck fractures in the elderly. Wang et al. conduct a cost-effectiveness analysis using a Markov decision model to compare HA and IF in treating nondisplaced femoral neck fractures in elderly patients in China. This study highlights that the HA is a cost-effective alternative to the IF for elderly patients with femoral neck fractures in mainland China.

For intertrochanteric fractures, implant failure remains one of the serious complications after surgery and the cut-out is one of the most common causes of internal fixation failure. For the stability of reconstructions after fracture reduction and fixation, 5 influencing factors have been summarized by Kaufer in 1980, that is, bone quality (osteoporosis), fragment geometry (comminution), fracture reduction quality, implant selection, and implant placement (TAD/Cal-TAD). The tip-apex distance (TAD) and the calcar-referenced tip-apex distance (Cal-TAD) have been suggested as the radiographic parameters that most predict the risk of cut-out. Barra and Barrios review a series 398 consecutive intertrochanteric fractures to check whether these two factors could predict implant cut-out treated by dynamic intramedullary nailing with the Trigen Intertan short nail included in a prospective study. According to this study, careful optimal reduction ensuring stable fixation with TAD >25 mm could reduce the occurrence of cut-out after dynamic intramedullary nailing of intertrochanteric fractures.

In summary, this research topic offers a comprehensive overview of recent advancements in new concepts and techniques to treat hip fracture. We extend our sincere gratitude to the authors for their rigorous research and to the reviewers whose contributions ensured the quality and relevance of this body of work. This collection serves as a testament to the progress in hip fracture surgery and a roadmap for future research aimed at enhancing the safety, efficacy, and personalization of hip fracture treatment.

